# Phylogeography and species distribution modelling of *Cryptocephalusbarii* (Coleoptera: Chrysomelidae): is this alpine endemic species close to extinction?

**DOI:** 10.3897/zookeys.856.32462

**Published:** 2019-06-17

**Authors:** Matteo Brunetti, Giulia Magoga, Mattia Iannella, Maurizio Biondi, Matteo Montagna

**Affiliations:** 1 Dipartimento di Scienze Agrarie e Ambientali, Università degli Studi di Milano, Via Celoria, 2, 20133 Milan, Italy Università degli Studi di Milano Milan Italy; 2 Dipartimento di Medicina clinica, Sanità pubblica, Scienze della Vita e dell’Ambiente, Università degli Studi dell’Aquila, Via Vetoio, 67100 Coppito, Italy Università degli Studi dell’Aquila Coppito Italy

**Keywords:** cold-adapted species, endemism, global warming, Italy, Orobie Alps, phylogeography, species distribution models, species extinctions

## Abstract

The alternation of glacial and interglacial cycles of the Quaternary period contributed in shaping the current species distribution. Cold-adapted organisms experienced range expansion and contraction in response to the temperature decrease and increase, respectively. In this study, a fragment of the mitochondrial marker COI was used to investigate the phylogeography of *Cryptocephalusbarii*, a cold-adapted alpine leaf beetle species endemic of Orobie Alps, northern Italy. The relationships among populations, their divergence time, and the most probable migration model were estimated and are discussed in light of the Pleistocene climate oscillations. Through a species distribution modelling analysis, the current habitat suitability was assessed and the distribution in a future global warming scenario predicted. The main divergence events that led to the actual population structure took place from ~750,000 to ~150,000 years ago, almost following the pattern of the climate oscillations that led to the increase of the connections between the populations during cold periods and the isolation on massifs in warm periods. The most supported migration model suggests that the species survived to past adverse climatic conditions within refugia inside and at the limit of the actual range. The species distribution modelling analysis showed that *C.barii* is extremely sensitive to air temperature variations, thus the increase of temperature caused by global warming will reduce the suitable areas within the species range, leading to its possible extinction in the next 50 years. *Cryptocephalusbarii* is a representative case of how cold adapted and limited distributed species have been and could be affected by climate change, that highlights the implementation of conservation actions.

## Introduction

The Quaternary Period, alternating at least seven glacial and interglacial cycles within the last 650,000 years, affected population migration and survival of animals and plants and thus contributed in shaping the current species distribution (Bennett 1990; Davis and Shaw 2001). During this period the average surface temperatures of Earth ranged from ~9 °C to ~16 °C compared to the present ~14 °C (Hansen et al. 2013). How European species adapted to warm and humid environments, both flora and fauna, have passed glacial cycles received a lot of attention in the last decades and it is documented that they survived glacial cycles in restricted areas of refugium represented by Balkan, Italian and Iberian peninsulas, which played a central role for the recolonisation of the temperate regions (e.g., Hewitt 1999, 2000). On the opposite, the interests towards understanding how cold-adapted species overcame glacial ages received limited attention (e.g., Schmitt 2009; Borer et al. 2010; Lohse et al. 2011). Warm- and cold-adapted organisms have shown opposite patterns in response to the decrease of the air temperature: the former shrank the range of distribution towards lower latitude during glacials (e.g., Hewitt 1999, 2000, 2004, 2011), while the latter likely expanded their range in the same periods (e.g., Muster and Berendonk 2006; Mardulyn et al. 2009; Lohse et al. 2011; Martinet et al. 2018). In detail, in the case of cold-adapted species, glacial cycles, associated with a decrease of temperatures, represented periods of range expansion towards lowland with possible contact among populations; when temperatures increased during interglacial periods, they suffered range shrink towards high-altitude lands. After the Last Glacial Maximum, mountainous reliefs of southern Europe (i.e., Balkans, Alps, Apennines, and Pyrenees) have represented the refugia for cold-adapted species, now inhabiting habitats at altitude higher than 1800 meters a.s.l. The interest on cold-adapted species is intended to increase since these taxa, usually consisting of limited and isolated populations, are currently suffering a dramatic shrink of their range, due to the present global warming (e.g., Dirnböck et al. 2011; Jacobsen et al. 2012; Rossaro et al. 2016). In this context, when cold-adapted species are endemic of a limited area, global warming could lead to their extinction (Malcolm et al. 2006; Urbani et al. 2015, 2017). In the last years, conservation policies have increased their effort towards the reduction of the biodiversity loss caused by global warming, but limited attention has been dedicated to the most species rich group of animals, the insects (Dunn 2005; Menéndez 2007).

Italy is an endemic species rich country both in term of flora and fauna (Peruzzi et al. 2014), in particular approximately the 10% of the fauna is composed by endemic species, mainly represented by invertebrates (Minelli et al. 2006). The north of Italy exhibits an especially high number of endemic species, the majority of them inhabit the southern margin of the Alps, where Pleistocenic refuges for cold-adapted species were present (e.g., Carapezza and Faraci 2006; Minelli et al. 2006; Tribsch and Schönswetter 2003; Negro et al. 2008). Besides animals, also plants show a high number of endemic taxa in the southern margin of the Alps (Cerabolini et al. 2004; Casazza et al. 2005; Peruzzi et al. 2014).

In this study we investigate the phylogeography of the alpine endemic leaf beetle *Cryptocephalusbarii* Burlini, 1948 (Coleoptera: Chrysomelidae) currently distributed on isolated areas of the southern part of the Alps (Orobie Alps), above 1,800 meters of altitude. The species was described by Burlini in 1948 basing on eight specimens collected in Alben Mount (Burlini 1955) and, beside it, reported also for Pizzo Arera, northern Grigna and Presolana. The species, on the basis of its morphological features, is considered part of *Cryptocephalussericeus* Linnaeus, 1758 species complex (Sassi 2014), but it is well distinguishable from the other species of the complex especially for the total black external habitus. As well as the majority of the species belonging to *C.sericeus* complex, it feeds on yellow flowers, in particular on *Hieraciumtenuiflorum*, *Leontodonautumnalis*, *Telekiaspeciosissima* (Asteraceae), and *Helianthemumnummularium* (Cistaceae) (Regalin and Redigolo 1993; Sassi 2014). The phylogenetic relationships of the species is controversial; on the basis of nuclear and mitochondrial markers *C.barii* results sister of the *sericeus* – *hypochaeridis* clade (Gómez‐Zurita et al. 2012), while on the base of a cladistic analyses performed on morphological characters it is the sister species of *Cryptocephalusatrifrons* Abeille, 1901 due to the presence of an upward plate replacing the lateral margin of the third endophallic sclerite (Sassi 2014).

In this study, through an extensive sampling across the distributional range of the species, we have investigated the phylogeography of *C.barii* in order to define the relationships between the currently isolated populations, estimate their divergence time taking into account Pleistocene climate oscillations, assess the current habitat suitability and predict the distribution of this orophilous and endemic species in a future global warming scenario.

## Materials and methods

### Sampling, DNA extraction, and PCR amplification

**Figure 1.**
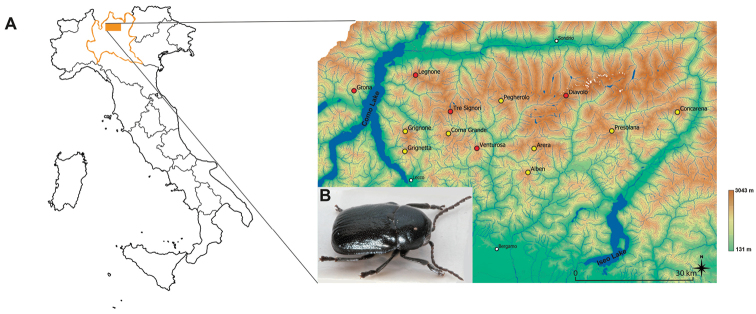
*Cryptocephalusbarii* Burlini, 1948 distribution. **A**) Geographic location of the Orobie Alps and *Cryptocephalusbarii* distribution. Yellow dots indicate localities where the species was observed, red dots indicate localities investigated with extensive sampling campaigns in which the species was absent (the source map was downloaded from http://www.geoportale.regione.lombardia.it/ and elaborated with QGIS 3.4.1). **B**) *Cryptocephalusbarii* picture acquired using a Canon 450D camera; the multilayered micrographs were processed with Zerene Stacker (Richland, WA, USA).

Between 2005 and 2012, different collecting campaigns were organised on mountainous reliefs of the Orobie Alps where the species was already known to be present, viz. Alben, northern Grigna, Presolana, and Arera (Burlini 1955). In addition, in order to investigate the distribution of *C.barii*, additional collecting campaigns were performed on mountainous reliefs surrounding the previous massifs and suitable for the presence of the species (i.e., elevation above 1800 meters and consisting of limestone) (Figure 1). Collected individuals were labelled with geographic coordinates, date and host plant on which they were found; then preserved in absolute ethanol and stored at –20 °C. With the exception of the Corna Grande population, where only two individuals were collected, seven to ten individuals from each population were selected for the DNA extraction. Total genomic DNA was extracted from each individual through the non-destructive procedure described in Montagna et al. (2013) and purified using the Qiagen DNeasy Blood and Tissue Kit (Qiagen, Hilden, Germany) following manufacturer’s instructions. The extracted DNA was quantified using NanoDrop2000 spectrophotometer and used as a template for PCR reactions. A fragment of the mitochondrial cytochrome c oxidase subunit I (COI), of approximately 730 bp, was amplified with primer C1-J-2183 / TL2-N-3014 (Simon et al. 1994). PCR were performed in 25 µL reaction mix containing: 1´ GoTaq reaction Buffer (10 mM Tris-HCl at pH 8.3, 50 mM KCl and 1.5 mM MgCl_2_), 0.2 mM of each deoxynucleotide triphosphate, 0.5 pmol of each primer, 0.6 U of GoTaq DNA Polymerase and 30 ng of DNA. PCR conditions used the following thermal cycle parameters: 3 min at 95 °C followed by 35 cycles of 30 s denaturation at 95 °C, 30 s annealing at 50 °C and 1 min 20 s extension at 72 °C, with a final single extra extension step of 10 min at 72 °C. PCR products were directly sequenced, in both strands, with ABI 3100 Automated Capillary DNA Sequencer (Applied Biosystem, Foster City, CA, USA). The obtained electropherograms were edited and assembled in a consensus sequence using Geneious Pro 5.3. The consensus sequences were deposited in GenBank with accession numbers MK492325-MK492374.

### Nucleotide variability, phylogeographic analysis, and divergence time estimation

The obtained 53 COI sequences were aligned using MUSCLE (Edgar 2004) with default parameters. The alignment was checked for reading frame errors and termination codons with MEGA 7 (Kumar et al. 2016). Intra-population and inter-population nucleotide p-distances were calculated with MEGA 7 (Kumar et al. 2016). In order to evaluate correlation between nucleotide and geographic distance matrices, the geographic distances between sampling locations were computed using R software (R Core Team 2016) starting from the latitude and longitude coordinates. Matrices correlation was estimated by Mantel test (Mantel 1967), as implemented in *ade4* R package (Dray and Dufour 2007).

Phylogeographic relationships within *C.barii* were investigated based on Bayesian inference using the software BEAST 2.5.1. (Bouckaert et al. 2014) with the bModelTest module (Bouckaert and Drummond 2017) for the evaluation of the substitution model. In addition, the dataset was tested for strict clock model against the non-clock model using a Bayes factor comparison. The marginal likelihoods of the two models were estimated by the stepping stone method implemented in MrBayes 3.2 (Ronquist et al., 2012), and then compared. According to the criterion reported in Kass and Raftery (1995), the strict clock model was preferred since the difference between the marginal likelihoods of the two models was not significant (p-value > 0.2). We used the alignment partitioned by codon positions, as suggested by preliminary analysis performed with Partition Finder 2.1.1 (Lanfear et al. 2016), and the strict clock model with a substitution rate set to 0.0177 ± 0.0003 My^-1^ (Papadopoulou et al. 2010), previously used for congeneric species (Montagna et al. 2016).

The tree prior was set using the Constant coalescent Kingman model (Kingman 1982). The analysis was carried out using a random starting tree, running two Markov chains for 100 million generations and sampling every 10,000 generations. Finally, the same analysis was performed sampling from priors only to evaluate the priors that we applied to the various parameters. Convergence was evaluated with Tracer version 1.7.1 (Rambaut et al. 2018), and the two chains were combined with LogCombiner (Drummond et al. 2012), discarding 20% of the trees as burn-in. The combined set of trees was summarised as a maximum clade credibility tree with TreeAnnotator (Drummond et al. 2012).

In order to confirm the rooting position of the tree, we carried out a preliminary phylogenetic reconstruction using an alignment consisting of the haplotype sequences of *C.barii* and orthologous sequences of five species mined from GenBank (i.e., *Cryptocephaluscristula* Dufour, 1843, *Cryptocephalusasturiensis* Heyden, 1870, *Cryptocephalusflavipes* Fabricius, 1781, *Cryptocephalusazurescens* Escalera, 1914 and *Pachybrachis* sp.; accession numbers: HE600320, HE600302, KJ765877, HE600310, HF947529) used as outgroups. In this phylogenetic reconstruction, performed on a dataset of 15 sequences, the tree prior was set using the Yule model (Yule 1925; Gernhard 2008), while other settings were the same of the previous analysis. In order to take into account for introgression and hybridisation phenomena a network of haplotypes was inferred through the minimum spanning network method as implemented in the software PopART 1.7 (Leigh and Bryant 2015).

### Gene flow models

We used the coalescent-based program MIGRATE-N 3.7 (Beerli and Palczewski 2010), which estimate past migration rates between populations, to test whether *C.barii* species survived ice ages in situ on ice-free nunataks or on large mountain of refuge at the periphery. It would be obviously impossible to evaluate every possible model of migration so we chose a small set of models in order to test our hypothesis. The following six migration models have been evaluated (Suppl. matreial 1: Figure S1): (A) directional migration from west to east, assuming a colonisation from the west; (B) directional migration from east to west, assuming a colonisation from the east; (C) directional migration from south to north, assuming a colonisation from southern refugia; (D) directional migration from north to south, assuming a colonisation from a possible northern refugium (Pegherolo); (E) a mixed model with colonisation from southern (Grigna) and northern (Presolana) refugia (Lohse et al. 2011); (F) a model, suggested by the phylogenetic analysis, with directional migration from Grigna to Alben, then to Pegherolo and Corna Grande and then to the others (Arera, Concarena and Presolana). We estimated the mutation scaled effective population size θ = *xN_e_µ* (*x* = 1, for mitochondrial DNA), where *N_e_* is the effective population size and *µ* is the mutation rate, as well as mutation scaled migration rates *M = m/µ*, where *m* is the immigration rate per generation. We used the marginal likelihood values approximated with thermodynamic integration to compare models with natural logarithm Bayes factor and to calculate the probability of each model (P(model_i_)=mL(model_i_)/∑_j_^n^mL(model_j_); Beerli 2012).

We used the sequence model of Felsenstein (1984) and random starting genealogy. A preliminary analysis was run with parameter values inferred by an F_st_-based method to obtain θ and *M* estimates that were used as initial values of that parameters in subsequent analysis. Prior distributions for θ and *M* were uniformly distributed with boundaries 0–0.1 and 0–50000, respectively. We performed four independent runs for each analysis, each consisting of a burn-in period of 25 million Markov chain Monte Carlo (MCMC) steps, followed by 100 million steps. Samples were recorded every 5,000 steps, resulting in a total of 20,000 recorded parameter values for each replicate. We used a static heating scheme with four chains with temperatures 1.00, 1.50, 3.00 and 1,000,000, in order to improve the estimation of marginal likelihood (Beerli 2009).

Since the suitable habitat of the species is currently between 1,800 and 2,100 meters of altitude, we hypothesise that during glacials the amount of areas with a suitable habitat increase, thus allowing the formation of corridors connecting the massifs where the species is present. The correspondence between the phylogeography of the species and possible corridors of suitable habitat connecting the different mountainous reliefs was evaluated building maps of the Orobie Alps highlighting areas above a certain altitude by QGIS 3.4.1 software (QGIS Development Team 2009). The average vertical thermal gradient used in this study is of 0.54–0.58 °C every 100 meters, as estimated for Alpine regions by Rolland (2003).

### Species Distribution Modelling (SDM)

A dataset of 35 presence localities was generated from GPS-precision field-recorded points for the target species *C.barii*. Nineteen bioclimatic variables were downloaded from the web repository Worldclim.org (Hijmans et al. 2005) at 30” spatial resolution and cut to the extent of the European Alps (sensu Biondi et al. 2013) through the ‘Extract by Mask’ tool in ArcMap 10.0 (ESRI, 2010). After this process, variables were tested for possible multicollinearity through the ‘Band Collection Statistics’ tool in ArcMap 10.0 (ESRI, 2010), a correlation matrix was calculated and variables’ pairs exceeding the Pearson’s r value of 0.85 were discarded (Elith et al. 2006; Iannella et al. 2018a; Iannella et al. 2018b).

For the modelling process, the ‘biomod2’ package (Thuiller et al. 2016) was used in R environment (R Core Team 2016). In particular, models for current and future climatic conditions were calculated through different sets of variables. Considering that many Global Climate Models (GCMs) are available for future climatic conditions, four GCMs were used in this paper, namely the BCC-CSM-1 (Wu et al. 2014), CCSM4 (Gent et al. 2011), IPSL (Marti et al. 2010) and the MIROC-CHEM (Watanabe et al. 2011). In particular, two scenarios of different radiative forcing were selected to observe the possible differences in conditions of medium and very high increase of radiative forcing, namely the 4.5 and the 8.5, for 2070.

Each species distribution model obtained from the different GCMs was processed through the MEDI algorithm (Iannella et al. 2017), a recent technique used to weight-average different predictions in one single model, thus avoiding predictions from one GCM and/or giving equal weight to models with low performances (see below).

In biomod2, Generalized Linear Models (GLMs, set with type = “quadratic”, interaction level = 3), Multiple Adaptive Regression Splines (MARS, set with type = “quadratic”, interaction level = 3), Generalized Boosting Model, also known as Boosted Regression Trees (BRT, set with number of trees = 5000, interaction depth = 3, cross-validation folds = 10) and Maxent (MaxEnt, set with maximum iterations = 5000) were selected as single modelling techniques (Phillips et al. 2006). Model was calibrated in current climatic conditions with the BIOMOD_Modelling function; all models’ performance were evaluated through the True Skill Statistics (TSS) (Allouche et al. 2006) and the Area Under the Curve (AUC) of the receiver operating characteristic curve (Phillips et al. 2006), with the initial 80% of the occurrence dataset used to calibrate the model and the remaining 20% for the validation. Then, the BIOMOD_EnsembleModelling function was used to obtain Ensemble Model for the target species, with the ‘wmean’ (weighted mean) algorithm used to merge each single model based on the respective performance scores. The BIOMOD_EnsembleForecasting function was further used to project the calibrated model to future climatic scenarios (Thuiller et al. 2016). A Minimum Convex Polygon (MCP) was generated on the basis of the presence data; all cartographic and spatial processes were managed in ArcMap 10.0 (ESRI, 2010).

## Results

### Species collection and ecological notes

The extensive sampling campaigns within the Orobie Alps and in neighbouring massifs suitable for the presence of *C.barii* (according with the proposed criteria, Materials and Methods), led to the collection of 60 individuals on eight massifs: the already known Mount Alben, Pizzo Arera, Presolana, and northern Grigna, with the addition of the newly discovered southern Grigna (hereafter reported as Grigna in association with the geographical neighbour northern Grigna), Corna Grande, Pegherolo, and Concarena (Figure 1). The performed extensive collecting campaigns make us to likely exclude the presence of *C.barii* on Mount Legnone, Mount Venturosa, Pizzo dei Tre Signori, Pizzo del Diavolo, and Mount Grona (Figure 1), mountainous reliefs presenting habitat suitable for the species and geographically close to previously known populations. All individuals were collected from mid-July to the end of August at an altitude ranging from 1,601 (only two samples, most likely transported by wind from higher altitudes) to 2,100 meters a.s.l., feeding or mating on *Heliantemum nummularium, Hieracium* spp. or *Telekiaspeciosissima.* The habitat of collection consists of grassland dominated by *Sesleriacoerulea* and *Carexsempervirens* attributable to habitat code *6170 Alpine and subalpine calcareous grasslands* (European Community Habitat’s Directive, 92/43/EEC).

### Phylogeography, divergence time estimation, and gene flow

**Figure 2.**
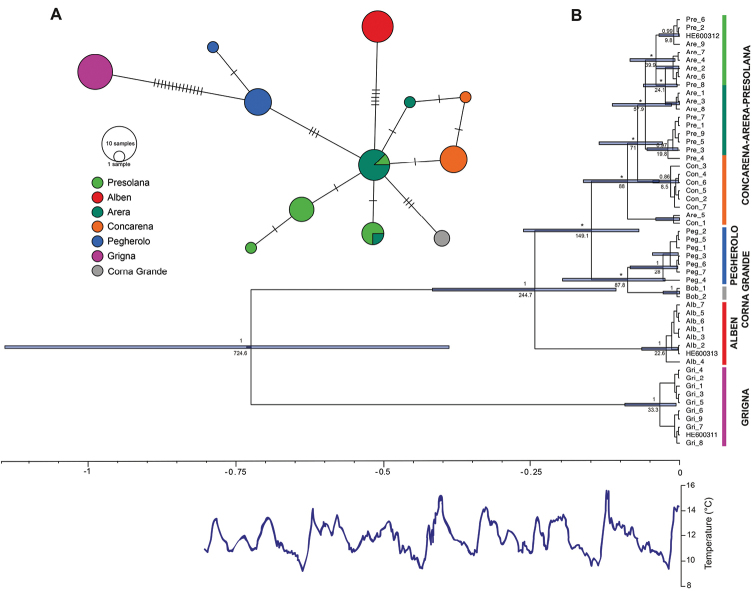
Maximum clade credibility tree and Minimum-spanning haplotype network. **A** Minimum-spanning haplotype network. Each colour represents a *Cryptocephalusbarii* population. Circles represent the different haplotypes; their diameter is proportional to the haplotypes abundance. **B** Maximum clade credibility tree. Horizontal blue bars represent 95% HPD age confidence intervals for the nodes. Under the main lineage nodes is reported the divergence time in thousands of years before present. Above the nodes Bayesian posterior probabilities > 0.8 are reported, black asterisks indicate Bayesian posterior probabilities values < 0.80. Vertical coloured bars on the right of the tree indicate monophyletic clades, the colours identify the *C.barii* populations. Under the tree, in blue, the estimated surface temperature for the last 800,000 years (Hansen et al. 2013).

DNA was extracted and COI amplified from 53 *C.barii* individuals (Table 1). Intra-population and inter-population mean pairwise nucleotide p-distance were 0.00047 (sd = 0.00064) [0–0.0018], and 0.011 (sd = 0.0083) [0.00049–0.024], respectively (Table 2). The population with the highest value of intrapopulation nucleotide p-distance was that of Presolana (0.0018, se = 0.0010), while the lowest values are associated with Alben, Corna Grande, and Grigna (0) (Table 2). Concerning the inter-population p-distance, the highest values were recovered comparing Grigna with other populations (mean p-distance = 0.023, sd = 0.0012), achieving the maximum value of 0.024 (se = 0.0057) when Grigna is compared with Alben, Corna Grande, and Concarena (Table 2). Some individuals collected in Presolana and Arera shared the same COI haplotypes, while all the other populations showed private haplotypes (Figure 2A). Positive correlation between geographic and nucleotide distances was detected by the Mantel test (r = 0.64, p-value = 0.001).

**Table 1.** Collection localities and host plants of *Cryptocephalusbarii* individuals from which the DNA was extracted, and COI amplified. Specimen IDs are reported for sequences obtained during this study, while for sequences already published in Gómez-Zurita et al. (2012) the accession numbers are provided.

Collection locality	Specimen id^†^	Collection date	Latitude	Longitude	E^‡^	Host plants
Alben	Alb-1	17 Aug 2010	45.8721N, 9.7807E		1855	*Helianthemumnummularium, Hieracium* sp., *Telekiaspeciosissima*
	Alb-2	08 Aug 2008	45.8714N, 9.7780E		1855	
	Alb-3	08 Aug 2008	45.8714N, 9.7780E		1855	
	Alb-4	17 Aug 2010	45.8723N, 9.7779E		1855	
	Alb-5	17 Aug 2010	45.8720N, 9.7766E		1855	
	Alb-6	17 Aug 2010	45.8727N, 9.7766E		1855	
	Alb-7	17 Aug 2010	45.8727N, 9.7766E		1855	
	HE600313	17 Aug 2010	45.8713N, 9.7787E		1855	
Arera	Are-1	23 Aug2010	45.9263N, 9.8013E		1929	*Helianthemumnummularium, Hieracium* sp.
	Are-2	23 Aug 2010	45.9176N, 9.7952E		1601	
	Are-3	23 Aug 2010	45.9309N, 9.8043E		2011	
	Are-4	23 Aug 2010	45.9309N, 9.8043E		2011	
	Are-5	23 Aug 2010	45.9336N, 9.8029E		2066	
	Are-6	23 Aug 2010	45.9336N, 9.8029E		2066	
	Are-7	23 Aug 2010	45.9176N, 9.7952E		1601	
	Are-8	23 Aug 2010	45.9263N, 9.8044E		1965	
	Are-9	23 Aug 2010	45.9401N, 9.8072E		2072	
Corna Grande	Bob-1	30 Jul 2011	45.9618N, 9.5206E		1900	* Helianthemum nummularium *
	Bob-2	30 Jul 2011	45.9618N, 9.5206E		1900	
Grigna (southern and northern Grigna)	Gri-1	17 Jul 2006	45.9663N, 9.3849E		1817	*Hieracium* spp., *Helianthemumnummularium, Telekiaspeciosissima*
	Gri-2	17 Jul 2006	45.9657N, 9.3870E		1817	
	HE600311	17 Jul 2006	45.9657N, 9.3870E		1817	
	Gri-3	17 Jul 2006	45.9657N, 9.3870E		1817	
	Gri-4	24 Jul 2011	45.9219N, 9.3763E		1700	
	Gri-5	1 Aug 2005	45.9649N, 9.3857E		1817	
	Gri-6	1 Aug 2005	45.9649N, 9.3857E		1817	
	Gri-7	1 Aug 2005	45.9649N, 9.3876E		1817	
	Gri-8	1 Aug 2005	45.9642N, 9.3856E		1817	
	Gri-9	13 Aug 2012	45.9218N, 9.3841E		1898	
Pegherolo	Peg-1	26 Aug 2010	46.0375N, 9.6929E		2100	* Helianthemum nummularium *
	Peg-2	26 Aug 2010	46.0358N, 9.6938E		2100	
	Peg-3	26 Aug 2010	46.0361N, 9.6953E		2100	
	Peg-4	20 Jul 2012	46.0351N, 9.6940E		2100	
	Peg-5	20 Jul 2012	46.0349N, 9.6940E		2100	
	Peg-6	20 Jul 2012	46.0349N, 9.6935E		2100	
	Peg-7	20 Jul 2012	46.0348N, 9.6943E		2100	
Presolana	Pre-1	11 Aug 2010	45.9696N, 10.0435E		2066	*Helianthemumnummularium, Hieracium* sp.
	Pre-2	11 Aug 2010	45.9640N, 10.0577E		1923	
	Pre-3	11 Aug 2010	45.9640N, 10.0577E		1923	
	Pre-4	11 Aug 2010	45.9655N, 10.0519E		2002	
	Pre-5	11 Aug 2010	45.9640N, 10.0577E		1923	
	Pre-6	11 Aug 2010	45.9640N, 10.0577E		1923	
	Pre-7	11 Aug 2010	45.9665N, 10.0494E		2031	
	HE600312	11 Aug 2010	45.9665N, 10.0494E		1938	
	Pre-8	11 Aug 2010	45.9665N, 10.0494E		2031	
	Pre-9	11 Aug 2010	45.9662N, 10.0531E		1995	
Concarena	Con-1	09 Sep 2012	46.0044N, 10.2735E		1750	* Hieracium tenuiflorum *
	Con-2	09 Sep 2012	46.0044N, 10.2735E		1750	
	Con-3	09 Sep 2012	46.0044N, 10.2735E		1750	
	Con-4	09 Sep 2012	46.0044N, 10.2735E		1750	
	Con-5	09 Sep 2012	46.0044N, 10.2735E		1750	
	Con-6	09 Sep 2012	46.0044N, 10.2735E		1750	
	Con-7	09 Sep 2012	46.0044N, 10.2735E		1750	

Note: †Specimen identifier; ‡Elevation is expressed in meters above sea level.

**Table 2.** Nucleotide p-distance within and between *Cryptocephalusbarii* populations.

**Comparison**	**Nucleotide p-distance^†^**
Alben	0 (0)
Arera	0.00066 (0.00045)
Corna Grande	0 (0)
Grigna (southern Grigna, northern Grigna)	0 (0)
Pegherolo	0.00042 (0.00041)
Presolana	0.0018 (0.0010)
Concarena	0.00042 (0.00040)
Alben – Arera	0.0074 (0.0032)
Alben – Corna Grande	0.0089 (0.0035)
Alben – Grigna	0.024 (0.0056)
Alben – Pegherolo	0.012 (0.0040)
Alben – Presolana	0.0080 (0.0033)
Alben – Concarena	0.0089 (0.0035)
Arera – Corna Grande	0.0045 (0.0026)
Arera – Grigna	0.022 (0.0055)
Arera – Pegherolo	0.0045 (0.0025)
Arera – Presolana	0.00049 (0.00048)
Arera – Concarena	0.0014 (0.0014)
Corna Grande – Grigna	0.024 (0.0058)
Corna Grande – Pegherolo	0.0059 (0.0029)
Corna Grande – Presolana	0.0050 (0.0026)
Corna Grande – Concarena	0.0059 (0.0029)
Grigna – Pegherolo	0.021 (0.0054)
Grigna – Presolana	0.023 (0.0055)
Grigna – Concarena	0.024 (0.0056)
Pegherolo – Presolana	0.0050 (0.0025)
Pegherolo – Concarena	0.0059 (0.0028)
Presolana – Concarena	0.0021 (0.0015)

Note: †Nucleotide p-distance: mean value and standard errors are reported, the latter within in brackets.

Based on the performed coalescence analysis, almost all the individuals from the same mountainous massif clustered together in monophyletic groups and are supported by high values of Bayesian posterior probability (BPP > 0.85; Figure 2B). However, in some cases, individuals from different massifs grouped together (Figure 2B); in details this phenomenon occurred for Arera and Concarena (two individuals; BPP = 0.92), Arera and Presolana (eight individuals; BPP < 0.5), Presolana and Arera (four individuals; BPP = 0.99) (Figure 2B).

Concerning the estimation of the divergence time among populations, the most ancient split, represented by the separation of Grigna lineage (tree rooted on outgroups) and all the remaining lineages occurred ~724,600 years before present (BP) (95% High posterior density (HPD) 1,140,700–389,900 years BP; BPP = 1) in correspondence with a period of warm climate, probably during the Pastonian or the Günz-Mindel interglacials (Figure 2B). The subsequent split, occurred about 244,700 years BP (95% HPD 417,900–107,500 years BP; BPP = 1) during the Mindel-Riss interglacial, determined the isolation of the Alben population and the next one, even if supported by values of posterior probability < 0.5, corresponded to the separation of Corna Grande-Pegherolo populations from individuals of Arera, Concarena, and Presolana. This last split is dated at about 149,100 years BP (95% HPD 263,900–68,900 years BP) corresponding to the Riss-Würm period, during the same interglacial the two populations of Corna Grande and Pegherolo separated one from the other ~87,800 years BP (95% HPD 197,700–24,500 years BP). The remaining populations (Arera, Concarena, and Presolana) diverged during the period from 162,700 to 83,700 years BP.

**Figure 3.**
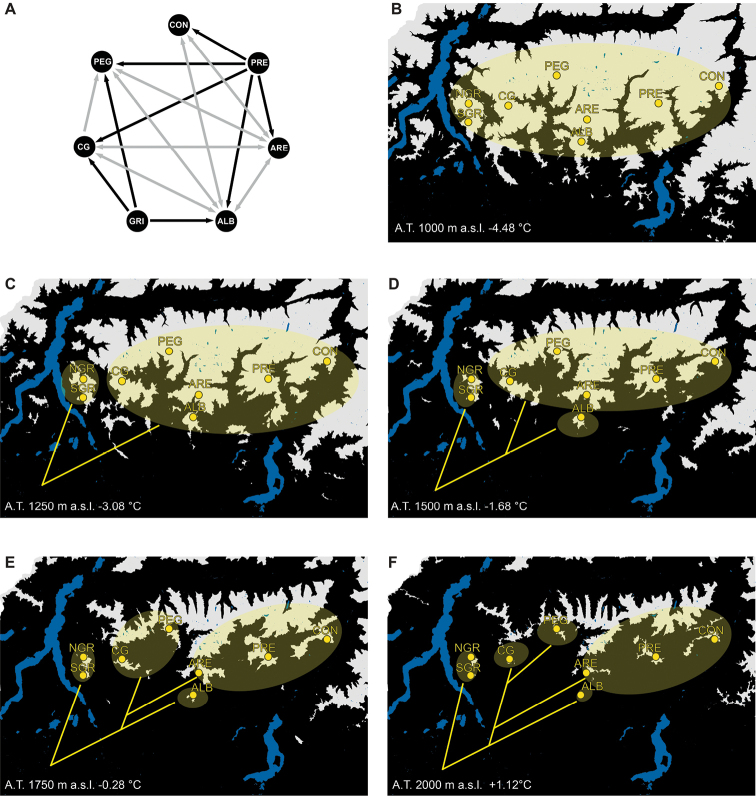
Most likely migration model and altitudinal habitat maps reporting suitable area for the presence of the species during cold periods. **A** Most likely migration model. Arrows indicate unidirectional flows (in black) and bidirectional flows (in gray) between populations. **B–F** Suitable altitudinal habitat maps. In light grey are reported the areas suitable for the presence of the species above a certain altitudinal threshold; the yellow ellipses are schematic drawing of *Cryptocephalusbarii* populations; dendrograms showing the divergence events among populations, according to the tree in Fig. 1, are superimposed on the maps. At the bottom left of each map are reported: the minimum elevation at which climatic conditions are suitable for the presence of *Cryptocephalusbarii* (inferred from present knowledge) corresponding to the altitudinal threshold used to draw the suitable areas, and the corresponding estimates of temperature variation in respect to the present. Abbreviations: A.T. = altitudinal threshold; CON = Concarena; PRE = Presolana; ARE = Arera; ALB = Alben; GRI = Grigna; NGR = northern Grigna; SGR = southern Grigna; CG = Corna Grande; PEG = Pegherolo.

In order to understand how *C.barii* populations became isolated on different mountainous reliefs, six possible migration models were formalised and tested (Suppl. material 1: Figure S1). Bayes factors, calculated as double the difference of natural log marginal likelihoods (LBF), between two competing models strongly supported the model (E) (LBFs > 5, Prob_model E_ = 0.908, Figure 3A). In this model, Grigna and Presolana are considered source population, according with the hypothesis that *C.barii* survived ice ages both on ice-free refugia in the inner core of its range and on large mountain of refuge at its southern periphery, with unidirectional gene flow directed toward other populations which instead exchange migrants bidirectionally.

The suitable altitudinal habitat maps, showing the increase of the areas appropriate for the *C.barii* survival and the available corridors connecting the present populations due to the decrease of the temperature, almost perfectly match with the topology achieved by the coalescent and the migration model analyses (Figure 3B–F). Interestingly, the populations inhabiting Arera, Presolana, and Concarena remained connected by habitat suitable for the species even when temperatures are only slightly lower than the present (Figure 3E). This last result is in agreement with the topology achieved by the phylogeographic analyses, where the relationships among these populations should be better represented by a polytomy or a network (BPP < 0.5; Figure 2).

### Species Distribution Modelling

**Figure 4.**
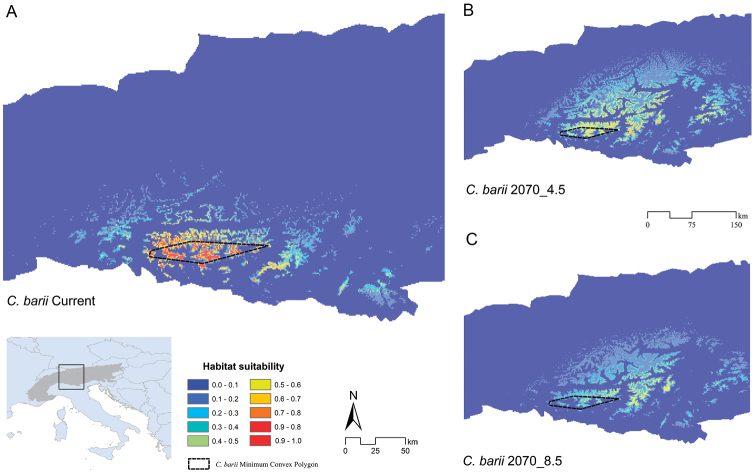
Predicted suitability for *Cryptocephalusbarii* for current and future climatic conditions. Predicted suitability resulting from the Ensemble Modelling process performed over bioclimatic variables for *Cryptocephalusbarii*, with the Minimum Convex Polygon built on the species’ presence sites for **A** current **B** 2070 – 4.5 scenario of radiative forcing, and **C** 2070 – 8.5 scenario of radiative forcing.

Concerning the Species Distribution Modelling (SDM) analysis, multicollinearity among the nineteen bioclimatic predictors was prevented by discarding nine variables, keeping as predictors for the modelling process: the mean diurnal range (BIO2); the isothermality (BIO3); the maximum temperature of the warmest month (BIO5); the minimum temperature of the coldest month (BIO6); the mean temperature of the wettest quarter (BIO8); the mean temperature of the driest quarter (BIO9); the annual precipitation (BIO12); the precipitation of the wettest month (BIO13); the precipitation seasonality (BIO15); the precipitation of the driest quarter (BIO17). The corresponding correlation matrix is reported in Suppl. material 2: Table S1. Ensemble Models (EMs) resulted in very high scores of TSS (0.998) and AUC (0.988); the ‘wmean’ maps resulting show, for current climatic conditions, a predicted suitable area which narrowly encompasses the known distribution range (represented by a Minimum Convex Polygon, MCP) (Figure 4A). The suitable climatic conditions are, in fact, strictly limited within *C.barii*’s MCP, with few areas with low suitability in the surrounding mountains, and no suitable areas in the valleys (Figure 4A). The most contributive bioclimatic variables were resulted: BIO3 (34%), BIO17 (27%), and BIO2 (14%).

**Figure 5.**
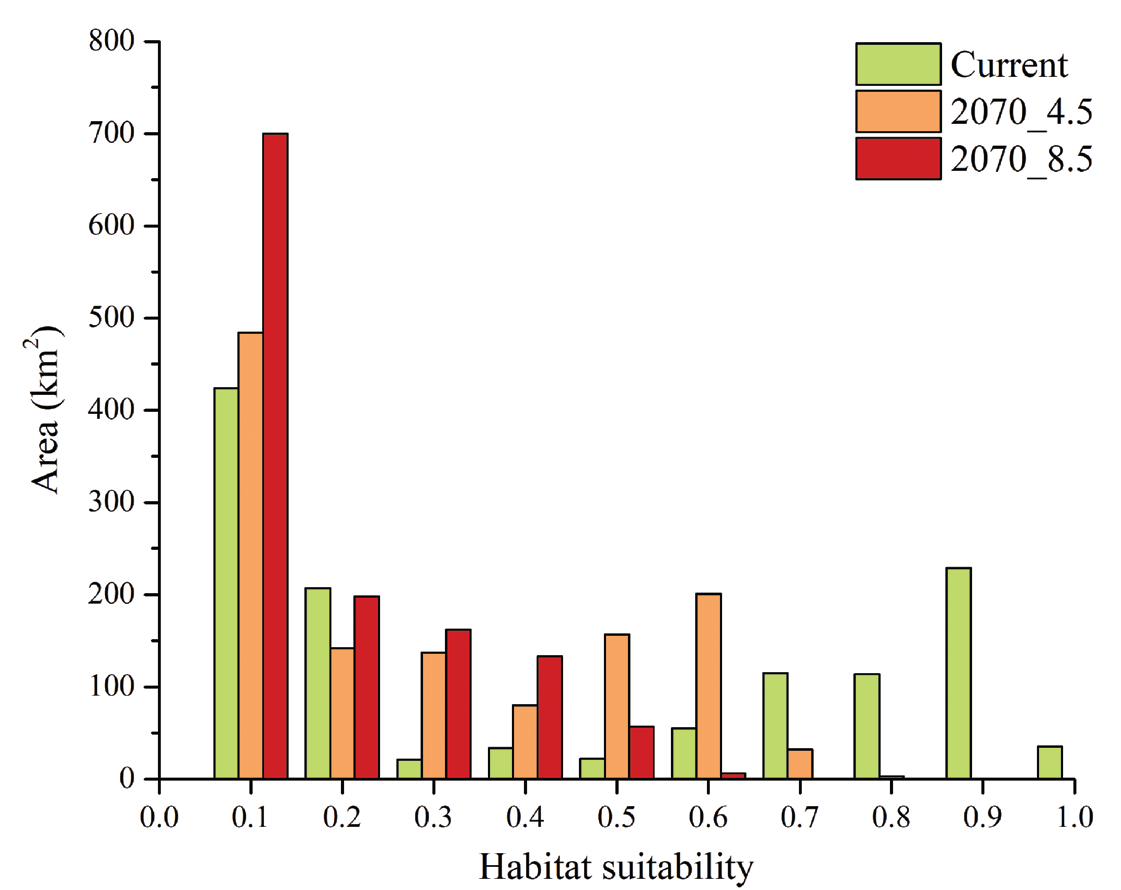
Changes in habitat suitability for *Cryptocephalusbarii*. Histogram reporting classes of habitat suitability calculated within the Minimum Convex Polygon built on *Cryptocephalusbarii* presence sites through Ensemble Modelling process. Areas calculated for current and future climatic conditions (2070, 4.5 and 8.5 scenarios) are reported, respectively, in green, orange and red.

For future scenarios, the 2070_4.5 predictions resulted in a partial north-eastern shift of the habitat suitability, with an apparent increase of the compatible areas, which however show lower suitability with respect to the current situation (Figure 4B), even in the MCP area (Figure 5). For the 2070_8.5 scenario, a general decrease of suitability is observable, even though a range expansion is predicted; nevertheless, also in this scenario the suitability in the MCP is much lower than the current climatic Ensemble Model (Figure 4C, Figure 5).

## Discussion

With this study the presence of the species is discovered on four mountainous reliefs from which it was never sampled before, thus extending its previous distribution towards the north (Pegherolo), east (Concarena), and south (southern Grigna). Since during the collecting campaigns the species has been searched also on suitable areas outside the previously known species range and it was not detected, we can be confident in supporting the fact that the species is nowadays confined in a limited area between the Como (on west) and Iseo (on east) lakes, corresponding to the glacial paleochannels of Adda and Oglio glaciers. The actual range of *C.barii* is limited to mountainous and geographically isolated calcareous reliefs of Orobie Alps, thus presenting a patchy distribution similar to that of an insular species inhabiting an archipelago. Regarding populations size, we observed that the most vigorous populations are those of northern Grigna (Circo di Moncodeno), Alben, Presolana, and Arera; while, those of southern Grigna, Pegherolo, Corna Grande, and Concarena inhabit a surface restricted area and consist of a limited number of individuals.

Species distribution modelling analyses showed that the most contributing variables retained as predictors are the mean diurnal range, the isothermality and the precipitation of the driest quarter. As other insect species associated with high altitude (Urbani et al. 2017), also *C.barii* shows a significant habitat suitability inversely proportional to the increase of isothermality values, indicating a high sensitivity to large and instable fluctuations in temperature. At present the habitat suitability within the current estimated range of the species (identified with MCP) is high, while in the case of the tested future scenarios the habitat suitability within the MCP suffered a decrease. Noteworthy, it has been predicted an increase of the compatible areas even if associated with low-intermediate values of habitat suitability, with a partial north-eastern shift of suitable areas. The results of our phylogeographic analyses showed a strong population structure, in accordance with previous studies on congeneric species, where a limited gene flow between populations were observed even when geographic distances are short (Piper and Compton 2003). Personal observations (M Montagna) on *C.barii* adult behaviour (individuals feeding or mating on a host plant once disturbed return back on it within few minutes) suggest a limited movement ability. These aspects, in association with the presence of biogeographical barriers, viz. the Como Lake on west, the Valtellina on north and the Val Camonica on east, delimiting the actual species range, let us to conclude that the possibility of the species to disperse and colonise new suitable areas within 50 years has to be considered highly improbable.

A geographic structure characterising *C.barii* populations was first confirmed by the positive correlation between geographic and nucleotide distances resulting from the Mantel test, even if based on a single mitochondrial marker. Indeed, most of the populations are characterised by private COI haplotype and only Presolana and Arera partially share haplotypes (Figure 2A). Furthermore, the reciprocal monophyly that characterise most of the populations analysed (Figure 2), even those geographically close such as Alben and Arera or Grigna and Corna Grande, suggests that such populations have been isolated from each other for a quite long period of time, and that the present population structure was only marginally affected by the last glaciation and by the Holocene climate optimum occurred between 9,000 and 5,000 years before present (Mayewski et al. 2004). The estimated time of divergence, even adopting a general nucleotide substitution rate, suggests that most of the divergence events largely predate the last glacial maximum in the Alps occurred approximately 26,500–23,500 years BP (Ivy-Ochs et al. 2008; Monegato et al. 2007). The isolation of the populations on separate mountains, occurred during warm periods when the species followed its optimal climate towards the higher quotes, was probably caused by ancient glacial cycles (from more than 1 million years ago to about 100,000 years BP). The last glacial cycle seems to have had limited effect in shaping the species population structures. Furthermore, the isolation of Grigna population (724,600 years BP) from the other populations largely predate the subsequent splits, represented by the divergence of Alben population (244,700 years BP) and Corna Grande-Pegherolo populations (149,100 years BP). The long-term isolation of the Grigna population is also supported by its high average nucleotide distance from other populations (0.0228 ± 0.0056), a value that is above the interspecific threshold inferred for the subfamily of Cryptocephalinae (1% nucleotide distance, Magoga et al. 2018). Grigna, Alben, Corna Grande and Pegherolo seem to be well isolated from other populations, while Arera, Presolana and Concarena, resulting not monophyletic and sharing haplotypes, are not genetically isolated. In detail, Arera and Presolana share two haplotypes and two COI sequences from Arera and Concarena cluster together in coalescent tree. This fact suggests the possibility that gene flow between these three populations occurred in the last 90,000 years.

The long-term isolation and the high average nucleotide distance from other populations inferred for individuals collected from Grigna seem to counteract with the migration model that has been selected as the most probable (Figures 2, 3A), indeed this model considers Grigna and Presolana as source population from which re-colonisation towards other mountains begun. At the same time, Presolana has the highest haplotype diversity and it is highly connected with at least two other populations (Concarena and Arera) making it justified to suppose that this could have act as a refugium during Holocene interglacials characterised by an increase of the temperature. Further analyses, based on the use of innovative approaches such as RAD (Restriction site Associated DNA) sequencing, have to be adopted in order to shed light on the past demography of this species and elucidate the mode and time of the migration processes occurred among populations.

## Conclusions

In this study, through an extensive sampling the comprehensive distribution of *C.barii*, species endemic of Orobie Alps, was defined. As expected, the population genetic structure of this cold adapted species was strongly affected by Pleistocene climate oscillations; in fact the observed phylogeographic patterns reflect population connections and isolation during cold and warm periods, respectively. Even if the obtained results are based on a single mitochondrial marker and not on the whole mitochondrial genome or part of the nuclear genome, the correspondence between presence of corridors among populations predicted at different temperatures and the observed genetic variability, let us to be confident about the accuracy of COI in phylogeographic pattern reconstruction, at least in the analysed case. This result further confirms the possibility to exploit the huge amount of COI sequences developed through DNA barcoding and DNA metabarcoding studies in the last 15 years, not only for DNA taxonomy purpose but also for phylogeography and genetic conservation ones.

The reduction of *C.barii* habitat suitability predicted within 50 years because of global warming, in association with the presence of biogeographic barriers that prevent the species dispersion, open the possibility that *C.barii* will be extinct during this time span. This prediction, in association with the observed low population size, the isolation of populations and the limited area of occupancy of the species prompt us to propose the inclusion of *C.barii* in the IUCN Red List as vulnerable or superior category, thus requiring urgent conservation actions pursued by Natural Parks and environmental agencies.

The case of *C.barii* can be representative of the cold adapted species, both animals and plants, currently present in the Alpine arc and inhabiting high altitude environments. Such species can be considered habitat specialists and the spatial extent of areas with suitable characteristics will be strongly reduced in the next years due to global warming. Beside the decrease in term of biodiversity, caused by the possible species extinctions, the impact on ecosystems, produced by the loss of these habitats, is currently unknown.

## Supplementary materials

Supplementary material 1 Migration models tested in this study using Migrate-n E) Most likely migration model Data type: statistical model Explanation note: Arrows indicate unidirectional flows (in black) and bidirectional flows (in gray) between populations. Abbreviations: con = Concarena; pre = Presolana; are = Arera; alb = Alben; gri = Grigna; cg = Corna Grande; peg = Pegherolo. This dataset is made available under the Open Database License (http://opendatacommons.org/licenses/odbl/1.0/). The Open Database License (ODbL) is a license agreement intended to allow users to freely share, modify, and use this Dataset while maintaining this same freedom for others, provided that the original source and author(s) are credited.Matteo Brunetti, Giulia Magoga, Mattia Iannella, Maurizio Biondi, Matteo Montagna

Supplementary material 2 Correlation matrix built among the 19 candidate predictors downloaded from the online repository Worldclim.org. Data type: statistical data Explanation note: Variables showing a Pearson’s | r | > 0.85 are highlighted in yellow and were discarded from the model building process. This dataset is made available under the Open Database License (http://opendatacommons.org/licenses/odbl/1.0/). The Open Database License (ODbL) is a license agreement intended to allow users to freely share, modify, and use this Dataset while maintaining this same freedom for others, provided that the original source and author(s) are credited.Matteo Brunetti, Giulia Magoga, Mattia Iannella, Maurizio Biondi, Matteo Montagna
