# Supplementary material for: Phylogeography and species distribution modelling of Cryptocephalusbarii (Coleoptera: Chrysomelidae): is this alpine endemic species close to extinction?
Source: Zookeys. 2019 Jun 17;856:3–25. doi: 10.3897/zookeys.856.32462 (PMC6603993; doi:10.3897/zookeys.856.32462)
Supplement: Supplementary material 1 [file zookeys-856-003-s001.docx]

Supplementary Table 1. Correlation matrix of the 19 candidate predictors.

|  | **BIO1** | **BIO2** | **BIO3** | **BIO4** | **BIO5** | **BIO6** | **BIO7** | **BIO8** | **BIO9** | **BIO10** | **BIO11** | **BIO12** | **BIO13** | **BIO14** | **BIO15** | **BIO16** | **BIO17** | **BIO18** | **BIO19** |
| --- | --- | --- | --- | --- | --- | --- | --- | --- | --- | --- | --- | --- | --- | --- | --- | --- | --- | --- | --- |
| **BIO1** | 1.00 | 0.73 | 0.03 | 0.60 | 0.98 | 0.92 | 0.69 | 0.60 | 0.53 | 0.99 | 0.97 | -0.72 | -0.74 | -0.67 | 0.04 | -0.75 | -0.65 | -0.68 | -0.57 |
| **BIO2** | 0.73 | 1.00 | 0.05 | 0.85 | 0.84 | 0.43 | 0.96 | 0.60 | 0.22 | 0.79 | 0.57 | -0.60 | -0.51 | -0.59 | 0.06 | -0.49 | -0.58 | -0.34 | -0.56 |
| **BIO3** | 0.03 | 0.05 | 1.00 | 0.03 | 0.04 | 0.03 | 0.04 | 0.02 | 0.02 | 0.04 | 0.03 | -0.03 | -0.03 | -0.03 | 0.99 | -0.03 | -0.02 | -0.02 | -0.02 |
| **BIO4** | 0.60 | 0.85 | 0.03 | 1.00 | 0.74 | 0.26 | 0.96 | 0.72 | -0.11 | 0.70 | 0.38 | -0.48 | -0.34 | -0.50 | 0.09 | -0.32 | -0.56 | -0.09 | -0.58 |
| **BIO5** | 0.98 | 0.84 | 0.04 | 0.74 | 1.00 | 0.82 | 0.83 | 0.65 | 0.44 | 0.99 | 0.90 | -0.72 | -0.72 | -0.69 | 0.05 | -0.72 | -0.67 | -0.61 | -0.61 |
| **BIO6** | 0.92 | 0.43 | 0.03 | 0.26 | 0.82 | 1.00 | 0.36 | 0.39 | 0.68 | 0.87 | 0.99 | -0.62 | -0.73 | -0.56 | 0.00 | -0.75 | -0.51 | -0.77 | -0.41 |
| **BIO7** | 0.69 | 0.96 | 0.04 | 0.96 | 0.83 | 0.36 | 1.00 | 0.68 | 0.06 | 0.77 | 0.50 | -0.57 | -0.46 | -0.57 | 0.08 | -0.44 | -0.60 | -0.24 | -0.60 |
| **BIO8** | 0.60 | 0.60 | 0.02 | 0.72 | 0.65 | 0.39 | 0.68 | 1.00 | -0.24 | 0.65 | 0.47 | -0.60 | -0.33 | -0.67 | 0.13 | -0.32 | -0.72 | -0.07 | -0.79 |
| **BIO9** | 0.53 | 0.22 | 0.02 | -0.11 | 0.44 | 0.68 | 0.06 | -0.24 | 1.00 | 0.46 | 0.65 | -0.26 | -0.54 | -0.17 | -0.08 | -0.55 | -0.05 | -0.76 | 0.12 |
| **BIO10** | 0.99 | 0.79 | 0.04 | 0.70 | 0.99 | 0.87 | 0.77 | 0.65 | 0.46 | 1.00 | 0.93 | -0.71 | -0.72 | -0.68 | 0.05 | -0.72 | -0.66 | -0.63 | -0.60 |
| **BIO11** | 0.97 | 0.57 | 0.03 | 0.38 | 0.90 | 0.99 | 0.50 | 0.47 | 0.65 | 0.93 | 1.00 | -0.67 | -0.75 | -0.62 | 0.02 | -0.77 | -0.57 | -0.76 | -0.48 |
| **BIO12** | -0.72 | -0.60 | -0.03 | -0.48 | -0.72 | -0.62 | -0.57 | -0.60 | -0.26 | -0.71 | -0.67 | 1.00 | 0.88 | 0.96 | -0.10 | 0.88 | 0.94 | 0.73 | 0.90 |
| **BIO13** | -0.74 | -0.51 | -0.03 | -0.34 | -0.72 | -0.73 | -0.46 | -0.33 | -0.54 | -0.72 | -0.75 | 0.88 | 1.00 | 0.76 | -0.02 | 0.99 | 0.71 | 0.92 | 0.64 |
| **BIO14** | -0.67 | -0.59 | -0.03 | -0.50 | -0.69 | -0.56 | -0.57 | -0.67 | -0.17 | -0.68 | -0.62 | 0.96 | 0.76 | 1.00 | -0.13 | 0.76 | 0.99 | 0.61 | 0.94 |
| **BIO15** | 0.04 | 0.06 | 0.99 | 0.09 | 0.05 | 0.00 | 0.08 | 0.13 | -0.08 | 0.05 | 0.02 | -0.10 | -0.02 | -0.13 | 1.00 | -0.02 | -0.14 | 0.01 | -0.15 |
| **BIO16** | -0.75 | -0.49 | -0.03 | -0.32 | -0.72 | -0.75 | -0.44 | -0.32 | -0.55 | -0.72 | -0.77 | 0.88 | 0.99 | 0.76 | -0.02 | 1.00 | 0.71 | 0.93 | 0.63 |
| **BIO17** | -0.65 | -0.58 | -0.02 | -0.56 | -0.67 | -0.51 | -0.60 | -0.72 | -0.05 | -0.66 | -0.57 | 0.94 | 0.71 | 0.99 | -0.14 | 0.71 | 1.00 | 0.52 | 0.97 |
| **BIO18** | -0.68 | -0.34 | -0.02 | -0.09 | -0.61 | -0.77 | -0.24 | -0.07 | -0.76 | -0.63 | -0.76 | 0.73 | 0.92 | 0.61 | 0.01 | 0.93 | 0.52 | 1.00 | 0.39 |
| **BIO19** | -0.57 | -0.56 | -0.02 | -0.58 | -0.61 | -0.41 | -0.60 | -0.79 | 0.12 | -0.60 | -0.48 | 0.90 | 0.64 | 0.94 | -0.15 | 0.63 | 0.97 | 0.39 | 1.00 |

Note: the correlation matrix built among the 19 candidate predictors downloaded from the online repository Worldclim.org. Variables showing a Pearson’s | r | > 0.85 are highlighted in yellow and were discarded from the model building process.
