# Supplementary figures and images for: Phylogeography and species distribution modelling of Cryptocephalusbarii (Coleoptera: Chrysomelidae): is this alpine endemic species close to extinction?
Source: Zookeys. 2019 Jun 17;856:3–25. doi: 10.3897/zookeys.856.32462 (PMC6603993; doi:10.3897/zookeys.856.32462)

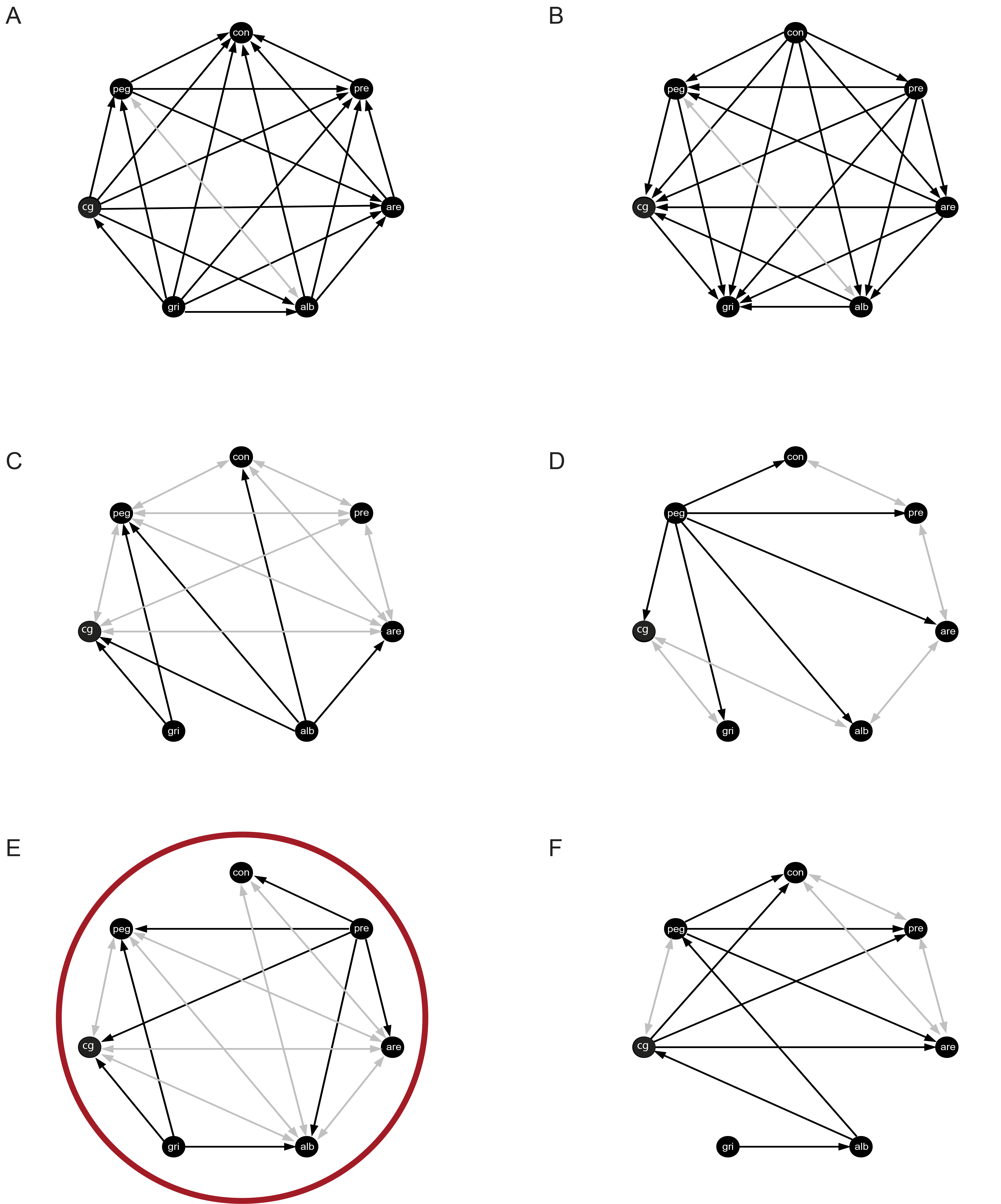

Supplement: Supplementary material 2 [file zookeys-856-003-s002.tif]
